# Review of Chemistry for Students

**Published:** 1851-03

**Authors:** 


*Review of Chemistry for Students. Adapted to the courses as taught in the Principal Medical Schools in the United States.* By John G. Murphy, M. D., Philadelphia. Lindsay & Blakiston, 1851.

This little compendium is one of the best condensations on the subject, with which we are acquainted, and we very cordially recommend it.

